# The Association Between Symptoms of Depression and School Absence in a Population-Based Study of Late Adolescents

**DOI:** 10.3389/fpsyg.2020.01268

**Published:** 2020-06-09

**Authors:** Kristin G. Askeland, Tormod Bøe, Astri J. Lundervold, Kjell M. Stormark, Mari Hysing

**Affiliations:** ^1^Regional Centre for Child and Youth Mental Health and Child Welfare, NORCE Norwegian Research Centre, Bergen, Norway; ^2^Department of Psychosocial Science, Faculty of Psychology, University of Bergen, Bergen, Norway; ^3^Department of Biological and Medical Psychology, Faculty of Psychology, University of Bergen, Bergen, Norway

**Keywords:** school absence, adolescence, depression, sleep duration, mental health problems

## Abstract

**Objective:**

School attendance is an important functional marker in adolescence, and knowledge of the correlates of school absence is important to inform preventive efforts. The main aim of the present study was to investigate the association between symptoms of depression and school absence in late adolescence, adjusting for sociodemographic characteristics and externalizing problems.

**Methods:**

Data stem from the youth@hordaland-survey, a population-based survey of adolescents between 16 and 19 years old attending upper secondary education in Hordaland County, Norway, in spring 2012. Administrative data on school absence was provided for 8222 adolescents. In addition to days and hours absent the past semester, a variable of total absence was calculated and divided into quartiles of absence. Symptoms of mental health problems and sleep duration was based on adolescent self-reports.

**Results:**

Reports of depressive symptoms were significantly associated with school absence when investigated as continuous variables. The strength of the association attenuated but remained statistically significant when controlling for sociodemographic factors and externalizing problems. When investigating the association at different levels of school absence, adolescents in the second, third and fourth quartile of school absence reported significantly higher depression scores compared to adolescents in the first quartile. The association between reports of symptoms of depression and school absence was partially mediated by sleep duration.

**Conclusion:**

The association between reported symptoms of depression and school absence was evident even at low levels of school absence, indicating a role for universal prevention strategies. The findings suggest both depression and sleep problems as possible targets for intervention in late adolescence.

## Introduction

The importance of school attendance has been emphasized in several studies. These studies show that continued absence from school is associated with poorer academic achievement (Carroll, 2010; Attwood and Croll, 2014) and predicts early school leaving and later unemployment and health problems (Freudenberg and Ruglis, 2007; De Ridder et al., 2012). These major individual and societal consequences of school absenteeism, in combination with high absence rates across countries, has led to increased focus on the topic. To prevent poor academic achievement, regular school attendance is essential and every day of absence can have negative consequences (Hancock et al., 2013). Gaining a better understanding of the correlates of absenteeism is therefore important to enable early identification of students at risk of school disengagement (Ingul et al., 2012).

School absenteeism is a complex phenomenon that is related to a range of individual, family and school characteristics (Kearney, 2008; Ingul et al., 2012; Gubbels et al., 2019). Mental health problems is one individual factor that has been consistently associated with absence both in clinical and community samples (Mcshane et al., 2001; Egger et al., 2003; Jones et al., 2009; Ingul et al., 2012; Finning et al., 2019a; Lawrence et al., 2019). Depression is described as one of the most important public health challenges in adolescence (Green et al., 2005; Hyde et al., 2008; Salk et al., 2016) and as an important risk factor for school absence (Jones et al., 2009; Wood et al., 2012; Skedgell and Kearney, 2016; Gonzálvez et al., 2018; Nayak et al., 2018; Finning et al., 2019a). This association was confirmed in a recent meta-analysis (Finning et al., 2019b). Further, both mild and severe depression have been implicated as important targets for interventions to improve school attendance (Gase et al., 2014). Though previous studies indicate a possible association between depression and school absence, many have focused on severe mental health problems (Mcshane et al., 2001; Egger et al., 2003) or adolescents with severe absenteeism referred to treatment or sent to court for their absence problems (Berg et al., 1993; Skedgell and Kearney, 2018). Further, the amount of school absence is often defined as problematic or non-problematic according to cut-offs based on expert consensus (Heyne et al., 2018), and few studies have investigated the association with depression across the distribution of school absence (Skedgell and Kearney, 2016). In order to target universal preventions aimed at promoting regular attendance for all students, we need to know what predicts absence at lower levels (not only above 15% which is often used as a cut-off). Using the multi-tiered approach suggested by Kearney and Graczyk (2014), this refers to interventions at tier 1, aimed at students with less than 5% absence.

An important consideration when assessing the association between depression and school absence is the influence of other co-occurring mental health problems, such as externalizing problems. Externalizing problems have consistently been shown to be associated with school absence (Ingul et al., 2012; Vaughn et al., 2013; Gubbels et al., 2019), and a recent meta-analysis found larger effect sizes for the association between school absence and externalizing problems than depression (Gubbels et al., 2019). Further, several studies have identified an overlap between internalizing and externalizing problems (Egger et al., 2003; Ingul et al., 2012), and this raises the question if co-occurring externalizing problems may account for the increase in absence related to depressive symptoms. The evidence base is conflicting. While one study found that co-occurring externalizing problems could account for the association (Ingul et al., 2012), there was still a significant association between depressive disorder and absence after controlling for externalizing problems in another study (Egger et al., 2003). Whether or not symptoms of depression are independently associated with school absence when controlling for externalizing problems will influence the choice of preventive efforts to reduce school absence and promote school attendance.

Furthermore, sleep problems should be taken into account (Finning et al., 2019b). Short sleep duration is not only frequent in adolescence (Hysing et al., 2013), it is both related to depression (Reigstad et al., 2010; van Zundert et al., 2015) and school attendance (Egger et al., 2003; Hysing et al., 2014) in this age group. A previous study based on the youth@hordaland found a significant association between sleep duration and school absence also when controlling for symptoms of depression (Hysing et al., 2014). It did not, however, investigate possible pathways. It is possible that sleep problems co-occur with mental health problems and school absence, but they may also be a pathway in which mental health problems manifest itself as school impairment.

Based on the above considerations, the main aim of the present study was to investigate the association between self-reported symptoms of depression and register-based school absence in a large population-based study in late adolescence. School absence will both be analyzed dimensionally as number of days and hours of absence, and as quartiles reflecting different levels of absence. Important covariates such as age, gender, parental education, economic well-being, and symptoms of externalizing problems will be adjusted for. If the association between symptoms of depression and school absence is significant, a possible indirect effect of sleep duration will be investigated.

## Materials and Methods

### Procedure

In this population-based study, we used data from the youth@hordaland-survey of adolescents in the county of Hordaland in Western Norway. All adolescents born between 1993 and 1995 and all students attending upper secondary education during spring 2012 were invited to participate. In Norway, compulsory educations ends at age 16, but adolescents between 16 and 19 years of age have a statutory right for upper secondary education. In 2012, 92% of all 16–18 year olds were registered in upper secondary education in Norway. The main aim of the survey was to assess prevalence of mental health problems and service use in adolescents.

Adolescents in upper secondary education received information via their school e-mail, and one classroom school hour was allocated for them to complete the questionnaire. The questionnaire was web-based and covered a broad range of mental health issues, daily life functioning, use of health care and social services, demographics, as well as a request for permission to obtain school data, and to link the information with national health registries. The Regional Centre for Child and Youth Mental Health and Child Welfare collaborated with Hordaland County Council to conduct the study.

### Sample

All adolescents born between 1993 and 1995 were invited (*N* = 19,430) to participate in the current study during the first months of 2012, of which 10,257 agreed, yielding a participation rate of 53%. Of these, 8988 adolescents consented to linkage to official school data provided by the Hordaland County Council and valid data on school absence was provided for 8222 adolescents.

### Ethics

The study was approved by the Regional Committee for Medical and Health Research Ethics (REC) in Western Norway. In accordance with the regulations from the REC and Norwegian health authorities, adolescents aged 16 years and older can make decisions regarding their own health (including participation in health studies). The adolescents thus gave consent themselves to participate in the current study. Parents/guardians have the right to be informed, and all parents/guardians received written information about the study in advance.

### Instruments

#### Demographic Information

Gender and date of birth was identified through the personal identity number in the Norwegian National Population Register. Exact age was estimated by calculating the interval of time between date of birth and date of participation. Socioeconomic status (SES) was assessed both by parental education and perceived economic well-being. Maternal and paternal education were reported separately with the response options: ‘primary school,’ ‘secondary school,’ ‘college or university: less than 4 years,’ ‘college or university: 4 years or more’ and ‘don’t know.’ The two categories pertaining to college or university education were combined into one, regardless of the length of the education. Economic well-being was assessed by asking the adolescents how they perceived the economic well-being in their family compared to most others. Response alternatives were ‘better than others, ‘equal to others,’ and ‘poorer than others.’

The educational programs reported by the adolescents were categorized into ‘general studies’ and ‘vocational studies.’ In the present study, only the adolescents with vocational subjects in a classroom setting were included in the latter category, excluding those in work placement where absence is not recorded by the schools. The categorization is based on the Norwegian upper secondary school system, which includes a program for general studies preparing for higher education and a vocational education program. Examples of vocational education tracks include Building and construction, Health care, childhood and youth development, and Information technology and media production.

#### School Attendance

Official register-based data on non-attendance were provided by Hordaland County Council and included separate variables for days and school-hours of absence from the last semester (6 months).

In addition to variables describing days and hours of school absence separately, *total school absence* was calculated by recoding hours of absence into days of absence based on the mandatory number of school hours each week in upper secondary school (an average of six school hours per day). Adding this new variable to the original variable of days of absence gave the definition of total absence. Total school absence was split into quartiles to investigate associations at different levels of absence.

#### Depressive Symptoms

Symptoms of depression were assessed using the short version of the Mood and Feelings Questionnaire (SMFQ). The SMFQ comprises 13 items assessing depressive symptoms rated on a three-point Likert scale. The wording of the response categories in the Norwegian translation equals the original categories of ‘not true,’ ‘sometimes true,’ and ‘true.’ High internal consistency between the items and a strong uni-dimensionality have been shown in population-based studies (Sharp et al., 2006), and confirmed by a study including a sample from the youth@hordaland (Lundervold et al., 2013). The standardized total SMFQ score (z-transformed) was used to indicate severity of depressive symptoms in the present study.

#### ADHD Symptoms

Symptoms of inattention and hyperactivity were assessed using the official Norwegian translation of the Adult ADHD Self-report Scale (ASRS) (Kessler et al., 2005). The questionnaire was originally constructed for use in adults, but has been validated for use among adolescents (Adler et al., 2012). ASRS is an 18 item self-report scale rated on a five-point Likert scale, comprising nine items assessing hyperactivity-impulsivity and nine items assessing inattention. The standardized total score across all 18 items was used to define severity of ADHD symptoms in the present study.

#### Symptoms of Conduct Problems

Symptoms of conduct problems were assessed using the Youth Conduct Disorder (YCD) scale (Lucas et al., 2001). The YCD consists of eight items with the response option ‘yes’ or’no,’ and the total number of ‘yes’ responses gives a measure of the severity of symptoms of conduct problems. It is part of the Diagnostic Interview Schedule for Children Predictive Scales (DPS), which has been shown to accurately identify adolescents with a high probability of meeting diagnostic criteria for conduct disorder (Lucas et al., 2001).

#### Sleep Duration

Self-reported bedtime and rise time were indicated in hours and minutes using a scroll down menu with 5 min intervals and were reported separately for weekdays and weekend. Time in bed (TIB) was calculated by subtracting bedtime from rise time. Sleep onset latency (SOL) and wake after sleep onset (WASO) were indicated in hours and minutes using an equal scroll down menu. Sleep duration was defined as TIB minus SOL and WASO.

### Statistical Analyses

To enable comparison across instruments, sum scores on the SMFQ, the ASRS and the YCD were standardized (z-transformed), with a mean of 0 and a standard deviation (SD) of 1. New categorical variables were created where the scores on each instrument were dichotomized at the 90th percentile, indicating the adolescents with the 10% highest scores. The 90th percentile was chosen as it is a well-established cut-off for dichotomizing between children and adolescents with and without risk of mental health problems (Goodman, 2001), and there are no official cut-off scores available for all the measures used in the present study. With regards to the SMFQ, a cut-off at 12 was suggested in a study of help-seeking adolescents aged 12–19 (Thabrew et al., 2018), while a previous study of Norwegian adolescents aged 10–19 used a cut-off at 11 (Larsson et al., 2016). The cut-off in the latter study corresponded roughly to the 90th percentile (Larsson et al., 2016). In the present study, the 90th percentile corresponds to a cut-off at 15, which is higher than in the previous studies. As the youth@hordaland is limited to late adolescence, higher scores is to be expected, in accordance with the findings of Larsson et al. (2016). Using the 90th percentile as a cut-off could be more inclusive than relying on the prevalence of mental disorders among Norwegian adolescents. We therefore conducted sensitivity analyses based on the 95th percentile, which yielded similar results to those relying on the 90th percentile.

Based on the scores dichotomized at the 90th percentile, we further created categorical variables identifying the adolescents scoring above the 90th percentile on (1) the SMFQ only, (2) one or both of the measures of externalizing problems (ASRS and YCD, but not the SMFQ) and (3) on both the SMFQ and one or both of the measures of externalizing problems (ASRS and YCD). Sensitivity analyses were conducted where the adolescents who scored above the 90th percentile on both the ASRS and the YCD were removed, which only led to small alternations in the numbers and did not affect the results notably.

Mean differences in absence according to gender and school program were investigated using independent samples *t*-tests. Due to the small number of participants who were 19 years old at the time of the survey (*n* = 221), 18- and 19-year-olds were allocated to the same age group. The effect sizes of the differences were calculated using the Cohen’s *d* formula and interpreted according to convention with d’s of about 0.2 representing small effect sizes, d’s of about 0.5 representing medium effect sizes and d’s of 0.8 and higher indicating large effect sizes (Cohen, 1988). Mean differences in absence according to age were investigated using analysis of variance (ANOVA), with eta-squared as a measure of effect size. Eta-squared describes the proportion of the total variation in the dependent variable that can be attributed to the independent variable (Fritz et al., 2012).

Due to excess zero-observations and over dispersion of the two outcome variables days and hours of absence, zero-inflated negative binominal (ZINB) regression was used to investigate the relationship between symptoms of depression and school absence. The ZINB regression analysis creates two separate models; a logit model for the zero-inflated part, predicting the likelihood of being a certain zero and the negative binominal model (count) that predicts the counts for participants who are not certain zeros. The two models are then combined. Average marginal effects (AMEs) were calculated to examine the expected increase in school absence following an increase of 1 SD in the independent variables in the negative binominal part of the model. In the zero-inflated part of the model, age, gender, parental education, and perceived economic well-being were included as predictors of having no absence. Odds ratios of the associations were calculated.

Multinomial logistic regression was used to investigate the association between symptoms of depression and quartiles of school absence. The quartiles of school absence were included as the independent variable and the first quartile, corresponding to the adolescents with the 25% lowest absence, was set as the reference group.

Three models were specified for the ZINB regression and multinomial logistic regression analyses. In the first two models, separate analyses were conducted with symptoms of depression, the total ASRS and YCD scores as independent variables. The associations with school absence were adjusted for age, gender, parental education, and perceived economic well-being in model 1. Preliminary analyses of age and gender as potential moderators of the association showed non-significant interactions, and they were therefore included as control variables in the analyses. In model 2, symptoms of depression, ADHD, and conduct problems were entered concurrently in the analysis, indicating their individual contributions.

The association between symptoms of depression and school absence was further examined by estimating a structural equation model allowing for mediation by sleep duration. The analysis was controlled for age, gender, parental education and perceived economic well-being, symptoms of ADHD and conduct problems. The robust maximum likelihood estimator was used, and missing data was handled by full information maximum likelihood (FIML). Indirect effects were investigated using the built-in function IND in Mplus. A significant mediation effect was determined using 95% bias-corrected bootstrap confidence interval. The standardized effect sizes are reported.

The independent samples *t*-test, ANOVA, ZINB regression, and multinominal logistic regression were conducted using STATA 15 (StataCorp, 2017). Mplus (version 8) (Muthén and Muthén, 1998–2017) was used for the mediation analysis.

## Results

### Sample Characteristics

The sample consisted of 8222 adolescents, 51.6% girls (see Table 1). The age of the participants ranged from 16 to 19 years and the mean age was 16.94 years (SD = 0.84 years). The most commonly reported education level was college/university for mothers (35.5%) and secondary school for fathers (34.6%). The majority of adolescents reported their economic well-being to be equal to others (65.7%), and few reported to be poorer than others (7.0%).

**TABLE 1:** Demographic characteristics of the sample.

	*n*	%	Range	% missing
Gender				
Female	4243	51.6		
Age [m(SD)]	16.94	0.84	16–19	0.1
*Parental education*				
Maternal education				1.45
Primary school	652	7.9		
Secondary school	2576	31.3		
College/university	2905	35.3		
Don’t know	1970	24.0		
Paternal education				1.68
Primary school	674	8.2		
Secondary school	2843	34.6		
College/university	2498	30.4		
Don’t know	2069	25.1		
Perceived economic well-being				2.69
Better than others	2029	24.7		
Equal to others	5399	65.7		
Poorer than others	573	7.0		
*Symptoms of mental health problems*				
Depression [m(SD)]	5.77	5.75	0–26	4.09
ADHD [m(SD)]	26.77	10.58	0–72	4.65
Conduct problems [m(SD)]	0.40	1.01	0–8	8.06

*m, mean; SD, standard deviation.*

### The Distribution of School Absence in the Sample

**FIGURE 1:**
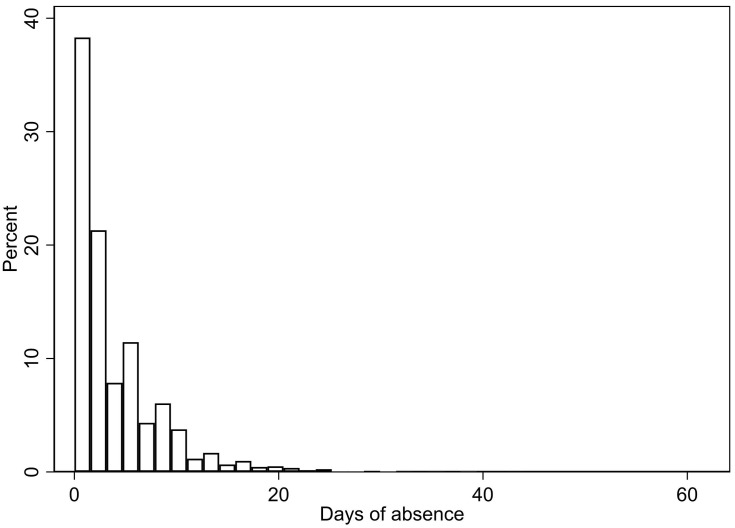
The distribution of school absence in days.

The distribution of school absence measured in days is shown in Figure 1, with a similar distribution for hours of absence. As detailed in Table 2, girls had a significantly higher number of days (*p* < 0.001, *d* = 0.20) and hours (*p* < 0.001, *d* = 0.11) absent than boys. Further, older adolescents had significantly higher absence compared to younger adolescents (*p* < 0.001, eta-squared = 0.03 for both days and hours of absence). Regarding school programs, there was a significant difference between adolescents attending general studies and vocational studies regarding days (*p* = 0.005, *d* = 0.07), but not hours (*p* = 0.864) of absence, with the highest school absence among adolescents attending vocational studies.

**TABLE 2:** School absence during one semester (84 school days) and demographic characteristics.

	Days of absence			Hours of absence		
		
	Mean (SD)	*p*-Value	Effect size	Mean (SD)	*p*-Value	Effect size
Gender		<0.001	*d* = 0.20		<0.001	*d* = 0.11
Boys	3.52 (4.76)			4.63 (8.65)		
Girls	4.54 (5.28)			5.66 (9.63)		
Age		<0.001	η^2^ = 0.03		<0.001	η^2^ = 0.03
16 (*n* = 2984)	3.40 (4.55)			4.00 (7.85)		
17 (*n* = 2991)	3.82 (4.69)			4.46 (8.22)		
18–19 (*n* = 2238)	5.22 (5.92)			7.89 (11.50)		
School program		0.005	*d* = 0.07		0.864	*d* = 0.006
General studies	3.86 (4.50)			4.98 (8.81)		
Vocational studies	4.19 (5.46)			5.03 (9.25)		

*SD, standard deviation; *d*, Cohen’s *d*; η^2^: eta-squared. Effect size for *t*-test: Cohen’s *d*, for ANOVA: eta-squared.*

### The Association Between Severity of Depressive Symptoms and School Absence

Results from the negative binominal part of the ZINB regression analysis showed a positive association between the total SMFQ score and days of school absence (see Table 3). Specifically, an increase of one standard deviation in the SMFQ score was associated with an increase in absence of 0.57 days (*p* < 0.001) when adjusting for demographic and socioeconomic variables. The associated increase in absence attenuated to 0.40 days (*p* < 0.001) when the ADHD and conduct problems symptom scores were included as covariates (model 2). Similar results were found for hours of absence, with an average marginal effect of 0.74 h of absence (*p* < 0.001) when adjusted for sociodemographic variables and 0.39 h (*p* = 0.004) when also adjusted for the ADHD and conduct problems symptom scores.

**TABLE 3:** The association between symptoms of depression and school absence in days and hours.

	Model 1				Model 2			
		
	Coef.	SE	*p*-Value	AME	Coef.	SE	*p*-Value	AME
**Days of absence**								
Depression	0.14	0.01	<0.001	0.57	0.10	0.02	<0.001	0.40
ADHD	0.14	0.01	<0.001	0.58	0.09	0.02	<0.001	0.38
Conduct problems	0.11	0.01	<0.001	0.46	0.08	0.01	<0.001	0.32
Age					0.20	0.02	<0.001	0.88
Gender					0.12	0.04	0.001	0.76
**Hours of absence**								
Depression	0.15	0.03	<0.001	0.74	0.08	0.03	0.004	0.39
ADHD	0.20	0.03	<0.001	0.99	0.15	0.03	<0.001	0.73
Conduct problems	0.16	0.03	<0.001	0.77	0.11	0.03	<0.001	0.51
Age					0.23	0.03	<0.001	1.97
Gender					0.05	0.06	0.423	0.61

*Coef., coefficient; SE, standard error; AME, average marginal effect. Model 1: adjusted for age, gender, parental education and economic well-being, Model 2: symptoms of depression, ADHD and conduct problems are entered concurrently and adjusted for the same covariates as in model 1. For gender, boy is set as reference.*

Results from the zero-inflated part of the model, predicting the likelihood of being a certain zero, were similar in all the analyses. Results from the fully adjusted model investigating associations with days of absence are presented. Older adolescents were less likely to have no absence compared to younger adolescents (OR = 0.67, 95% CI 0.50–0.91, *p* = 0.010), and girls were less likely to have no absence compared to boys (OR = 0.22, 95% CI 0.07–0.65, *p* = 0.006). Neither maternal education, paternal education nor perceived economic well-being were significantly related to the odds of having no absence (results not shown).

To investigate school attendance among adolescents with high scores on the SMFQ, the analyses were rerun using the dichotomous variables used to define high scorers on depression, ADHD symptoms and conduct problems. Adolescents scoring above the 90th percentile were compared to those with lower scores on the respective instruments. Results were similar to the analyses using the continuous scores, but the expected increases of absence both measured as days and hours were somewhat larger (see Table 4). In the fully adjusted model, scoring in the 90th percentile of depressive symptoms was related to an expected increase in absence by 1.09 days (*p* < 0.001). The corresponding increase in hours of absence was 1.28 h (*p* = 0.006).

**TABLE 4:** The association between high symptom scores of depression (90th percentile) and school absence in days and hours.

	Model 1				Model 2			
		
	Coef.	SE	*p*-Value	AME	Coef.	SE	*p*-Value	AME
**Days of absence**								
Depression	0.30	0.05	<0.001	1.38	0.25	0.05	<0.001	1.09
ADHD	0.34	0.04	<0.001	1.57	0.28	0.05	<0.001	1.26
Conduct problems	0.35	0.05	<0.001	1.61	0.27	0.05	<0.001	1.21
Age					0.20	0.02	<0.001	0.89
Gender					0.18	0.03	<0.001	0.97
**Hours of absence**								
Depression	0.31	0.08	<0.001	1.74	0.24	0.09	0.006	1.28
ADHD	0.45	0.08	<0.001	2.75	0.34	0.09	<0.001	1.93
Conduct problems	0.47	0.09	<0.001	2.83	0.38	0.09	<0.001	2.20
Age					0.24	0.03	<0.001	2.04
Gender					0.11	0.06	0.054	0.93

*Coef., coefficient; SE, standard error; AME, average marginal effect. Model 1: adjusted for age, gender, parental education and economic well-being, Model 2: symptoms of depression, ADHD and conduct problems are entered concurrently and adjusted for the same covariates as in model 1. For gender, boy is set as reference.*

### The Association Between Depression and Different Levels of School Absence

The associations between symptoms of depression and different levels of school absence were investigated by dividing the total absence into quartiles. The distribution of absence in quartiles is shown in Table 5.

**TABLE 5:** The distribution of total school absence in quartiles.

	*N*	Mean	SD	Min	Max	% absent
1st quartile	2827	0.39	0.47	0	1	0.5
2nd quartile	1740	2.34	0.54	1.17	3	2.8
3rd quartile	1670	4.74	0.84	3.17	6	5.6
4th quartile	1985	11.78	6.38	6.17	63	14.0

*SD, standard deviation. The column % absent includes the percent of the total number of school days in the semester the mean absence refers to.*

Compared to adolescents in the first quartile, i.e., with the 25% lowest absence, adolescents in the other quartiles had a higher relative risk for a higher SMFQ score in model 1 (see Table 6). There was a tendency of higher risk ratios in the higher quartiles, for instance, in model 1, there was a RRR of 1.11 (95% CI 1.03–1.20) in quartile 2 and a RRR of 1.41 (95% CI 1.33–1.51) in quartile 4 compared to quartile 1. The relative risk ratios of higher depression scores were slightly attenuated, but remained significant at all quartiles when the symptoms of ADHD and conduct problems were included in the analysis in model 2.

**TABLE 6:** The association between symptoms of depression and different levels of school absence (divided into quartiles).

	2nd quartile		3rd quartile		4th quartile	
			
	RRR (95% CI)	*p*-Value	RRR (95% CI)	*p*-Value	RRR (95% CI)	*p*-Value
*Model 1*						
Depression	1.11 (1.03–1.20)	0.004	1.22 (1.14–1.31)	<0.001	1.41 (1.33–1.51)	<0.001
ADHD	1.07 (1.00–1.14)	0.044	1.21 (1.13–1.29)	<0.001	1.46 (1.37–1.55)	<0.001
Conduct problems	1.13 (1.05–1.22)	0.001	1.21 (1.12–1.30)	<0.001	1.37 (1.28–1.47)	<0.001
*Model 2*						
Depression	1.09 (1.01–1.19)	0.028	1.13 (1.04–1.23)	0.003	1.24 (1.15–1.34)	<0.001
ADHD	1.03 (0.95–1.11)	0.509	1.12 (1.03–1.21)	0.006	1.30 (1.20–1.40)	<0.001
Conduct problems	1.10 (1.02–1.19)	0.019	1.16 (1.07–1.25)	<0.001	1.26 (1.18–1.36)	<0.001
Age	1.23 (1.13–1.34)	<0.001	1.43 (1.31–1.55)	<0.001	1.74 (1.60–1.89)	<0.001
Gender	1.23 (1.07–1.41)	0.001	1.38 (1.20–1.59)	<0.001	1.72 (1.49–1.98)	<0.001

*RRR, relative risk ratio. School absence divided into quartiles, with the 1st quartile as the reference group. Model 1: adjusted for age, gender, and parental education, Model 2: symptoms of depression, ADHD, and conduct problems are entered concurrently and adjusted for the same covariates as in model 1. For gender, boy is set as reference.*

### The Association Between School Absence and High Scores on Either Depression, Externalizing Problems, or Both

To further investigate the importance of symptoms of depression relative to externalizing problems for school absence, associations were investigated for adolescents scoring above the 90th percentile of the SMFQ only, adolescents scoring above the 90th percentile on measures of externalizing problems (ASRS or YCD) only and adolescents who scored above the 90th percentile on both the measures of depression and externalizing problems (see Table 7). Compared to adolescents with absence in the first quartile, adolescents in the higher quartiles had significantly higher relative risk of scoring above the 90th percentile on only depression and only externalizing problems. Regarding high scores on both depression and externalizing problems, significant differences were only found for the third and fourth quartile.

**TABLE 7:** The association between school absence (divided into quartiles) and high scores on either depression, externalizing problems, or both.

	2nd quartile		3rd quartile		4th quartile	
			
	RRR (95% CI)	*p*-Value	RRR (95% CI)	*p*-Value	RRR (95% CI)	*p*-Value
Depression	1.42 (1.05–1.92)	0.024	1.46 (1.08–1.98)	0.015	2.28 (1.73–2.99)	<0.001
Externalizing problems	1.47 (1.19–1.82)	<0.001	1.64 (1.32–2.03)	<0.001	2.64 (2.17–3.21)	<0.001
Both	1.09 (0.72–1.65)	0.798	1.73 (1.19–2.52)	0.004	3.08 (2.21–4.28)	<0.001
Age	1.22 (1.13–1.32)	<0.001	1.41 (1.30–1.53)	<0.001	1.72 (1.59–1.86)	<0.001
Gender	1.27 (1.12–1.44)	<0.001	1.44 (1.26–1.64)	<0.001	1.85 (1.62–2.10)	<0.001

*RRR, relative risk ratio. School absence divided into quartiles, with the 1st quartile as the reference group. Here, depression refers to adolescents scoring above the 90th percentile on the SMFQ only, externalizing problems refers to adolescents scoring above the 90th percentile on the ASRS, the YCD or both, and both refers to adolescents scoring above the 90th percentile on both the SMFQ and one of the measures of externalizing problems. For gender, boy is set as reference.*

### Sleep Duration as a Mediator

**FIGURE 2:**
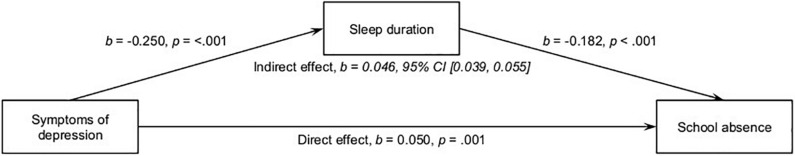
Sleep duration as a mediator on the association between symptoms of depression and school absence.

A mediation analysis was conducted to explore the effect of sleep duration on the association between symptoms of depression and school absence. As shown in Figure 2, the association was partially mediated by sleep duration, with a significant direct effect from symptoms of depression to school absence, as well as an indirect effect through shorter sleep duration with an increasing sum score on the symptom scale of depression.

## Discussion

In the present population-based study, depressive symptoms were significantly associated with number of days and hours of school absence among adolescents in upper secondary education in Norway. The association was attenuated by the presence of externalizing problems, but the depression score remained an independent predictor of school attendance. There was a trend toward stronger associations in the subgroup with high scores on the depression scale (scoring above the 90th percentile). When investigating different levels of school absence, adolescents with absence in the second, third and fourth quartiles has a significantly higher risk of more severe symptoms of depression. The association between severity of depressive symptoms and school absence was partially mediated by sleep duration.

The positive association between the total score on the depression scale and school absence is in line with previous studies and a recent meta-analysis (Egger et al., 2003; Jones et al., 2009; Wood et al., 2012; Skedgell and Kearney, 2016; Nayak et al., 2018; Finning et al., 2019a,b; Lawrence et al., 2019). The associations attenuated, but remained significant after adjusting for severity level of symptoms of externalizing problems, in line with previous research based on diagnosed disorders (Egger et al., 2003). However, it is in contrast to a previous study of Norwegian adolescents, where internalizing problems were no longer significantly associated with absence when accounting for externalizing problems (Ingul et al., 2012). This may be explained by differences in methodology and the inclusion of anxiety in addition to depression in the measure of internalizing problems in the previous Norwegian study (Ingul et al., 2012). In the study investigating diagnosed disorders, many of the internalizing disorders were no longer significantly associated with absence when controlled for comorbidity, only separation anxiety and depression predicted absence independently (Egger et al., 2003). Previous studies have found that associations with absence are stronger for symptoms of depression than anxiety (Jones et al., 2009; Finning et al., 2019a), and many adolescents with symptoms of anxiety continue to attend school on a regular basis (Ingul and Nordahl, 2013). It has been suggested that it could be more difficult for adolescents suffering from depressive symptoms to attend school regularly (Finning et al., 2019a), probably due to the specific symptoms of depression. It is likely that lack of energy, loss of motivation and difficulties concentrating could influence schoolwork and results. This could in turn lead to low self-esteem and feelings of hopelessness, increasing the likelihood that the adolescent does not attend school. Depression has been related to work-life participation among adults, especially among younger adults (Mykletun et al., 2006), and the symptoms showing the strongest association with work impairment include sad mood, problems concentrating, energy loss and sleep onset insomnia (Fried and Nesse, 2014).

The adolescents with the highest absence rates had the strongest association with symptoms of depression. This is in line with previous studies where high absence, defined as absence above 15% of school days, is related to an increase in internalizing problems that is not seen at lower levels of absence (Ingul et al., 2012; Skedgell and Kearney, 2016). Further, in a large study from the United States, adolescents who were classified as high level school skippers (above 13%) were more likely to report a history of depression, while no such association was found for adolescents with moderate absence (1–3 days) (Vaughn et al., 2013). Still, the present study detects significant differences in symptoms of depression between adolescents in the first and second quartile, groups with a mean absence of 0.5 and 2.8%, respectively. Thus, the present findings suggest that the associations are present also at lower levels of absence, supporting the notion that every day counts, also in the context of mental health problems.

Of note, the participants with school absence in the second quartile did not have a higher risk of having high scores on both depression and externalizing problems. Thus, co-existence of the two did not emerge as an important factor for those with a moderate level of school absence. A possible explanation is that while comorbid mental health problems is not an early warning sign of school absence, high scores on either symptoms of depression or externalizing problems are.

Interestingly, the association between reports of depressive symptoms and school absence was partially mediated by sleep duration. Absence has previously been associated with short sleep duration (Hysing et al., 2014) and sleep problems (Egger et al., 2003), but this is the first study to investigate the mediating role of sleep duration on the well-established association between symptoms of depression and school absence in a population-based sample. It appears that sleep duration could be one of the mechanisms by which depressive symptoms negatively influences school absence. A longer sleep onset latency that drives the short sleep duration in this age group is also a key characteristic of adolescents with depressive symptoms (Sivertsen et al., 2014) and may be closely related to ruminations at bedtime (Slavish and Graham-Engeland, 2015). Short sleep duration impairs coping with stressful situations in adolescence, thus shorter sleep related to depressive symptoms may impede coping with everyday school life and attendance (Wang and Yip, 2019).

Girls had significantly higher absence than boys both in days and hours, and older adolescents had higher absence than younger adolescents, which is consistent with previous research (Hancock et al., 2013; Maynard et al., 2017; Gubbels et al., 2019). We also know that both sleep duration and depressive symptoms are age and gender specific (Hysing et al., 2013; Lundervold et al., 2013; Maslowsky and Ozer, 2014). Still, there was no evidence of moderation, i.e., that the association differed between boys and girls and the different age groups. The finding regarding gender is in line with a recent meta-analysis (Gubbels et al., 2019) and a study of United Kingdom children (Finning et al., 2019a). In contrast to the present study, the latter study identified a significant moderating effect of age indicating a stronger association between depression and school absence for adolescents compared to children (Finning et al., 2019a). This is likely due to the different age compositions, where the previous study compared results in 5- to 11-year-old children to results in 11- to 16-year-old adolescents. As the age range is narrower in the present study, focusing solely on late adolescence, it appears that the associations remain stable in late adolescence.

### Strengths and Limitations

This is one of the first studies to investigate associations between symptoms of depression and absence both dimensionally and at different levels of absence in a large population-based survey. Strengths of the study include the large sample size and the use of an official registry of school absence. It is to the best of our knowledge the first study including sleep duration as a mediator on the association between depression and school absence.

A central limitation of the study is its cross-sectional nature, making it impossible to specify the direction of the associations. This is a challenge in the mediation analysis, and the results must therefore be interpreted with caution. In the mediation model, we assume that symptoms of depression precede school absence. This is based on previous longitudinal investigations which suggest that depression precedes school absence, especially in adolescence (Wood et al., 2012; Burton et al., 2014).

We were further not able to distinguish between different types of absence based on form or function in the present study (Heyne et al., 2018). A recent meta-analysis indicated that the association between depression and school absence differed according to the type of absence (Finning et al., 2019b), and it is thus possible that we would have more nuanced findings if such information was available. Furthermore, we were only able to include risk factors related to the adolescent in the present study, and do not have information on factors related to the family, school or peer group that could be important in understanding school absence (Kearney, 2008; Gubbels et al., 2019).

It is important to note that the diagnostic criteria for depression, ADHD and conduct problems were not included in the present study and scoring above the 90th percentile does not indicate fulfilling the diagnostic criteria for the respective mental disorders.

The response rate of the survey was only 53%, which limits the generalizability of the results. It is possible that adolescents experiencing more mental health problems were less likely to answer the questionnaire. Further, as the youth@hordaland-survey was school-based, it is likely that the majority of the participants completed the questionnaire during the allocated classes at school even though they could complete the questionnaire at their own convenience during the data collection period. Thus, adolescents without attendance problems were more likely to participate in the survey. Still, though no national statistics of absence rates are available for comparison, comparing the GPA in the sample to national statistics indicate that the sample is representative of adolescents attending school in terms of academic functioning (Hysing et al., 2016). Though the low response rate could bias the prevalence estimates of both mental health problems and school absence in the present study, it has been suggested that measures of associations are less affected by selective participation (Wolke et al., 2009).

### Implications

The present findings support the notion that every day of absence counts, also for mental health problems. Though this is increasingly recognized, and early intervention is emphasized, there is currently no consensus as to how much absence is too much absence and when to intervene (Chu et al., 2019; Ingul et al., 2019). In the present study, the association between severity of depressive symptoms and school absence was evident even at low levels of absence. This is important information for teachers and health personnel working with adolescents and suggest a role for universal prevention in schools. Universal prevention strategies are aimed at the entire population and aim to reduce a risk factor by a small amount for everyone, rather than restricting the strategies to those at high risk for mental health problems (Gordon, 1983; O’Connell et al., 2009). Since the present study reports mental health problems even at low levels of absence, a small reduction in mental health problems in the general adolescent population could contribute to reduce absence and related negative consequences. School based programs to prevent depression in adolescence have yielded a reduction in depression with small effect sizes (Werner-Seidler et al., 2017). It is possible that such a small reduction, if it concerns many adolescents, could significantly reduce absence. As we cannot know the direction of the associations based on the present cross-sectional data, it is further possible that increasing school attendance among adolescents could impact their mental health favorably in a similar manner. Still, the possibility of universal prevention and early interventions focused on emerging depression, sleep problems and school absence remain as speculations in the present study, and future research is needed to develop and validate intervention strategies. Indeed, the lack of studies focusing on evidence based prevention and early intervention strategies is identified as a gap in the current literature (Tonge and Silverman, 2019).

In addition to informing prevention strategies, the present study points to some early risk indicators that could be included in early assessments of students showing signs of emerging school absenteeism. The independent association of depression on school absence after accounting for externalizing problems, and the role of short sleep duration as a mediator suggests that measures to prevent school absence focused at the individual level could include both these problem areas. While symptoms of emotional distress are often included in such evaluations, sleep problems could be a valuable addition.

The results further point to several interesting avenues for future research. Firstly, the importance of sleep problems in school attendance merit further investigation and including absence as an outcome measure should be considered in intervention programs. Secondly, although the present study suggests associations between symptoms of depression and emerging school absence, longitudinal analyses are needed to further investigate their timing and order.

## Conclusion

The present study confirms that the association between symptoms of depression and school absence is found even at lower levels of absence in a population-based sample. The inclusion of sleep duration as a mediator is a valuable contribution to the literature, and our results indicate that short sleep duration could be one of the mechanisms linking symptoms of depression to school absence in adolescence.

## Data Availability Statement

The datasets for this manuscript are not publicly available due to privacy restrictions in accordance with the ethical approval for the youth@hordaland-survey. Requests to access the datasets should be directed to the Bergen Child Study group, bib@norceresearch.no.

## Ethics Statement

The studies involving human participants were reviewed and approved by the Regional Committee for Medical and Health Research Ethics (REC) in Western Norway. Written informed consent from the participants’ legal guardian/next of kin was not required to participate in this study in accordance with the national legislation and the institutional requirements.

## Author Contributions

MH, TB, and KA contributed to conception and design of the study. AL, KS, and MH contributed to data collection. KA performed the statistical analyses and wrote the first draft of the manuscript. All authors contributed to manuscript revision, read and approved the submitted version.

## Conflict of Interest

The authors declare that the research was conducted in the absence of any commercial or financial relationships that could be construed as a potential conflict of interest.
